# Sunlight-Induced Synthesis of Non-Target Biosafety Silver Nanoparticles for the Control of Rice Bacterial Diseases

**DOI:** 10.3390/nano10102007

**Published:** 2020-10-12

**Authors:** Hongyi Shang, Zehao Zhou, Xuemin Wu, Xuefeng Li, Yong Xu

**Affiliations:** Innovation Center of Pesticide Research, Department of Applied Chemistry, College of Science, China Agriculture University, Beijing 100083, China; hongyishang@cau.edu.cn (H.S.); zhouzh@nanoctr.cn (Z.Z.)

**Keywords:** silver nanoparticles, antibacterial, antibacterial mechanism, bacterial disease, biosafety

## Abstract

Silver is an important and efficient bactericide. Nanoscale silver has a large specific surface area, high target adhesion, strong permeability and high bactericidal activity. At present, the control of plant bacterial diseases is difficult, and the resistance of plant bacterial pathogens develops rapidly. Silver nanoparticles are expected to become a new generation of agrochemical to control plant bacterial diseases. In this study, a simple and green natural sunlight-induced method was used to prepare carboxymethylcellulose sodium-stabilized silver nanoparticles (CMC-SNs) with a particle size of around 13.53 ± 4.72 nm. CMC-SNs were characterized by dynamic light scattering (DLS), transmission electron microscopy (TEM), energy-dispersive spectrometry (EDS), X-ray diffraction (XRD) and UV-vis spectroscopy and found to be spherical and evenly dispersed. The bacteriostatic activity of the CMC-SNs toward *Xanthomonas oryzae* pv. *oryzae* (*Xoo*) was tested. The minimum inhibitory concentration (MIC) of CMC-SNs to *Xoo* was 1 mg/L, and the minimum bactericidal concentration (MBC) was 2 mg/L. In addition, the antibacterial mechanism was studied by scanning electron microscope (SEM) and confocal laser scanning microscope (CLSM), which confirmed that the CMC-SNs had high antibacterial activity. In order to verify its impact on the environment, we conducted an acute toxicity test on zebrafish and found that Half lethal concentration (LC_50_) > 100 mg/L in zebrafish, or no acute toxicity. The ability of CMC-SNs to control rice bacterial blight was verified by a pot experiment.

## 1. Introduction

At present, plant bacterial diseases cause huge economic losses in the world every year. The management of bacterial diseases is a troublesome issue and challenge for plant protection [1]. As the global cultivated land area is decreasing and the global population is increasing, we need to produce safe and high-yield food continuously and reliably. One of the main concerns is the control of bacterial diseases. In China, only 2.6% of the total registered fungicide products are used to control bacterial diseases. However, the annual occurrence area of bacterial diseases in China is around 8 million hectares, and the annual occurrence area of rice bacterial blight alone is nearly 0.27 million hectares. There is a big gap between demand and the development status of the industry. Copper compounds introduced in the 1880s and streptomycin introduced in the 1950s are the main agents used to control plant bacterial diseases. However, numerous plant bacterial pathogens have developed different levels of resistance to copper compounds and streptomycin [2,3]. One of the most widely used antibiotics, streptomycin was officially withdrawn from the Chinese market in 2016, because of the threat of large-scale drug resistance and effects in human. As a result, there is a vacancy in the pesticide economy for controlling bacteria in agriculture. Researchers are committed to finding a new, efficient, low-cost and low-resistance pesticide for killing agricultural bacteria.

Silver and silver salts have been used by human beings since ancient times. They have been used as disinfectants for 1200 years [4]. Thus far, silver has been proven to have broad-spectrum antibacterial effects in clinical medicine. As early as the 1970s, silver was proven to be the element most toxic to microorganisms, followed by Hg, Cu, Cd, Cr, Pb, Co, Au, Zn, Fe, Mn, Mo, Sn [4]. According to research, the bacteriostatic ability of the silver ion is slightly higher than that of silver nanoparticles [5], but at the same time, the damage of silver ions to plasma membranes and proteins makes it highly toxic to humans, the environment and other organisms. In Ancient China, metallic silver products have been used to make chopsticks, hairpins and rings, which can directly contact the human body without toxicity, and have a bactericidal effect, which is beneficial to human health. Therefore, metallic silver is safe for humans, other organisms and the environment. At the nanoscale, the particles show better performance and have larger specific surface areas, which means that they can contact bacterial cells more easily [6]. At the same time, the smaller size also means that they may be toxic at the cellular, subcellular and molecular biological levels, so the toxicity and environmental impact of nanomaterials should be studied.

Nano-silver has a larger specific surface area; thus, nano-sliver particles are capable of penetrating the cell membrane or attaching to the bacterial surface based on their smaller size, so their bactericidal activity, permeability and transmissibility are better than streptomycin and copper compounds [2]. Currently, there are two main explanations for the antibacterial mechanism of silver nanoparticles [7,8,9,10]. The first is that nano-silver contacts the bacterial cell wall, which is deformed and atrophied, and destroys the structure of the bacterial cell wall and cell membrane, causing the outflow of substances in a bacterial cell. The second proposed mechanism is that nano-silver enters the cell interior, combines with proteins to destroy its structure, and produces reactive oxygen species (ROS) to attack the plasma membrane, protein and DNA, leading to cell death. The antibacterial efficiency is increased by lowering the particle size [9]. Electrostatic forces that develop when nanoparticles with a positive zeta potential encounter bacteria with a negative surface charge promote a closer attraction and interaction between the two entities and possibly the penetration into bacterial membranes [8]. Different from the antibacterial mechanisms of streptomycin and copper compounds, only acting on the protein structure [2], nano-silver has a unique antibacterial mechanism which may reduce the occurrence of drug resistance.

According to statistics, the research and application fields of silver nanoparticles mainly focus on biocides, food contact materials, feed additives and food additives [11]. Up to now, the application of nano-silver particles in plant disease control has mainly focused on fungal diseases [2]. Silver nanoparticles have many excellent characteristics, which may make them an excellent bactericide in the future. However, the limitations of their cost, preparation, particle dispersion and ingredient delivery still need to be addressed. In addition, before large-scale application, it is necessary to determine the environmental behavior of silver nanoparticles and the toxicity of non-target organisms.

The production of metal nanoparticles in a natural environment is due to the reduction of metal ions in a water environment by the dissolved organic matter under the catalysis of sunlight [12]. Compared with the common synthesis methods of silver nanoparticles, such as chemical synthesis [13], light discharge [14] and biosynthesis [15,16,17], this method is simple, green and produces no by-products. In this study, silver nanoparticles were prepared from easily available raw materials by a natural sunlight-induced method, and as-prepared silver nanoparticles were characterized by transmission electron microscopy (TEM), dynamic light scattering (DLS), X-ray diffraction (XRD), energy-dispersive spectrometry (EDS) and UV-vis spectroscopy. The antibacterial activity of these silver nanoparticles on *Xanthomonas oryzae* pv. *oryzae* (*Xoo*) and the two mechanisms described above were studied. The non-target biosafety of the prepared silver nanoparticles was evaluated by an acute toxicity test on zebrafish. Through the pot experiment of *Oryza sativa L.* (rice), it was shown that the CMC-SNs have good prospects in the application of rice bacterial disease control.

## 2. Materials and Methods

### 2.1. Materials and Instruments

Carboxymethyl cellulose sodium (300–800 mPa·s), silver nitrate and ascorbic acid were purchased from Sinopharm Chemical Reagent Co. Ltd (Shanghai, China); all other reagents were of analytical grade. All the aqueous solutions were prepared with distilled and deionized water.

The instruments used in this work included the following: a magnetic stirrer from SILE (Shanghai, China), an incubator from PEIYING (Suzhou, China), a UV-vis spectrometer from SHIMADZU (UV-1800, Kyoto, Japan), a scanning electron microscope (SEM, Hitachi S-4800, Tokyo, Japan), a transmission electron microscope (TEM, FEI Tecnai G2 F30, Hillsboro, OR, US), a laser particle size analyzer (Malvern Zetasizer nano ZS, Malvern, UK), an X-ray powder diffractometer (XRD, Xpert pro MDP, PANalytical B.V., Almelo, Netherlands) and a confocal laser scanning microscope (CLSM, Leica TCS SP8 CARS, Berlin, Germany).

### 2.2. Natural Sunlight-Induced Synthesis of CMC-SNs

First, 1% carboxymethyl cellulose sodium (CMC-Na) was diluted to make the pre-solution, and 20 mM silver nitrate was slowly added to the pre-solution dropwise and stirred continuously at room temperature. The solution was exposed to light with a cumulative photosynthetically active radiation of 66.7 W/m^2^ for 3.0 h. Under the sunlight, the color of the solution gradually changed from colorless to brown, and the reaction was complete after 3 h. Lastly, two drops of ascorbic acid were added to clear the unreacted silver ions.

### 2.3. Characterization and Analysis

TEM with EDS was used to observe the morphology and particle size of the metallic silver nanoparticles. The EDS mode was applied for elemental composition analysis. The specific method involved dispersing the nanoparticles in the aqueous phase and then dropping them onto the Cu grid. Sample images were captured at 300 kV. ImageJ software was used to manually measure the particle diameter in TEM. The software version was 1.52 v.

DLS was further used to evaluate the size of the particles. Measurement parameters were as follows: a measurement temperature of 25 °C, a medium viscosity of 0.8872 mPa·s and a dispersant refractive index of 1.330, material refractive index of 1.59.

The progress of the reaction and optical properties of silver nanoparticles was analyzed by using UV-vis absorption spectrophotometry. Slit width 1 nm, the wavelength range was 300 to 800, sampling interval was 0.2 s.

XRD was used to determine the crystallinity and morphology. The samples were centrifuged and dried to obtain solid powder, which was analyzed by X-ray powder diffractometer. The XRD pattern was analyzed with a detector voltage of 40 kV and a current of 200 mA using CuKα radiation (*λ* = 1.54056 Å). The recorded range of 2θ was 20–80.

SEM was used to study the mechanism by which the bacterial cell wall was destroyed by CMC-SNs. The acceleration voltage was 3 kV, and the magnification was ×40.0k to ×70.0k. The vacuum system was a turbomolecular pump.

CLSM was used to study the bactericidal mechanism of nano-silver on bacteria. The scanning speed was 4000 line/s, and the output speed was 4000 MHz. The resolution of spectral scanning was 2 nm, the step was 1 nm, and the scanning speed was 100 nm/1 msec. The excitation/emission wavelength was 488/516 for SYTO9 and H2DCFDA and 535/617 for PI.

### 2.4. Antibacterial Activity

The main bacteria studied in this experiment were *Xanthomonas oryzae* pv. *oryzae* (provided by the College of Plant Protection, China Agricultural University), which are the pathogenic bacteria of rice bacterial blight. The bacteria were resuscitated and cultured in broth medium in a shake-flask at 150 rpm for the following test. In this method, the numbers of viable bacterial cells were analyzed using the colony forming units (CFU); the bacterial suspension was diluted to around 1 × 10^8^ CFU/mL (0.5 McFarland standard) as a mother liquor, which was determined by optical density at 600 nm (OD_600_) [18]. The mother liquor was diluted with a liquid medium, and the initial concentration of bacteria in each shake-flask was around 10^5^ CFU/mL. CMC-SNs with gradient concentration were added into each flask, and the concentration gradient was set as 4, 2, 1, 0.5, 0.25, 0.125, 0.0625, 0.03125 mg/L, and the untreated group was set as the control. Bacteria were cultured at 28 °C for 12 h. MIC, the minimal inhibitory concentration to effectively prevent the growth of bacteria in vitro, was used to evaluate the result of antibacterial activity. The antibacterial activity of silver ions was compared to the antibacterial activity of CMC-SNs. The concentration gradient of silver ions was consistent with CMC-SNs.

Streptomycin was selected for comparison. The effective concentration range of streptomycin for *Xoo* was determined. The concentration gradient of *Xoo* was set to 206.7, 172.8, 144, 120, 100, 83.3 and 69.4 mg/L. The control group was not treated.

In order to further determine the antibacterial ability of CMC-SNs, two methods were used to analyze the treated bacterial solutions quantitatively. UV-vis spectrophotometry was used to analyze the treated bacterial suspension. The absorbance value and the concentration of bacteria are related. Therefore, bacterial survival rate can be determined, and LC_50_ value can be calculated. However, we found that the addition of high concentrations of CMC-SNs may affect the relationship between the absorbance and the number of bacteria, so we chose a second method to quantify the treated group of bacteria.

The second method was to dilute the treated culture medium, inoculate it on an agar plate and test the minimum bactericidal concentration (MBC) of CMC-SNs by the method of colony count. No bacteria survived on the agar plate, indicating that all bacteria were killed.

The antibacterial activity of CMC-SNs toward *P. syringae* pv. *panici* (Elliott) Young et al. (ATCC19860) and *Pseudomonas syringae* pv. *syringae* Van Holl (ATCC19875) was also examined. These are the pathogenic bacteria of *Pseudomonas avenae* Manns and rice bacterial sheath rot, respectively.

### 2.5. Morphological Analysis

For the mechanism of nano-silver inhibiting bacteria, there are several opinions at present. Silver nanoparticles contact with the bacterial cell wall and destroy the permeability of the bacterial cell membrane by binding with proteins and allowing intracellular substances of the bacterial cells to leak out, leading to cell death [19]. Silver nanoparticles enter bacterial cells to destroy protein and DNA structures and produce oxygen free radicals to destroy the cell structure further, causing bacterial death [20]. In this paper, these two mechanisms were studied and verified. Firstly, the morphology of bacteria treated by CMC-SNs was analyzed. The treated bacterial solution was centrifuged, the supernatant was discarded, and the bacterial cells were resuspended and fixed with glutaraldehyde solution. After rinsing, dehydration, air drying and other steps, scanning electron microscopy was performed.

### 2.6. Membrane Permeability Analysis

Two fluorescent dyes, SYTO9 and PI, were selected to dye the treated bacterial cells in order to study the effect of nano-silver on cell membrane permeability. SYTO9 can dye the living bacteria green through the living bacterial cell membrane, while PI can only dye the genetic material of bacteria red through a destroyed cell membrane. The bacterial solution was diluted to 1 × 10^5^ CFU/mL by UV-vis spectrophotometer, and then CMC-SNs were added to break the bacterial cell membrane. Then, we dropped 10 μL of SYTO9 and PI solution. Therefore, the treated and untreated bacteria were centrifuged, resuspended, dyed, washed three times and resuspended. The change in bacterial cell membrane permeability was measured by laser confocal microscopy for treated and untreated cells.

### 2.7. Analysis of Oxygen Free Radicals (ROS) in Bacterial Cells

Silver nanoparticles enter the interior of bacteria and produce oxygen free radicals (ROS), which destroy the cell structure and lead to bacterial death. The production of ROS was demonstrated using an active oxygen probe, 2’,7’-dichlorodihydrofluorescein diacetate (H2DCFDA), which can penetrate into cells and react with ROS to form 2’,7’-dichlorofluorescein (DCF), which shows green fluorescence. The bacterial solution was diluted to 1 × 10^5^ CFU/mL by UV-vis spectrophotometer, and then CMC-SNs were added to break the bacterial cell membrane. Then, we dropped 10 μL of H2DCFDA. The treated and untreated bacteria were centrifuged, resuspended, dyed, washed three times and resuspended. The production of ROS in bacteria was observed by laser confocal microscopy.

### 2.8. Safety Evaluation to Non-Target Organisms

Materials at nanoscale often have a smaller particle size and larger specific surface area, which will show better performance. However, the safety of nanomaterials is a concern. As a heavy metal, silver salts are toxic to organisms, so the safety of CMC-SNs must be considered. The CMC-SNs prepared in this study are mainly used to control rice bacterial diseases, so the safety of non-target organisms such as fish could pose a significant problem. Research shows that bare silver nanoparticles have acute toxicity to zebrafish [21]. The CMC-SNs prepared in this study are encapsulated by sodium carboxymethylcellulose, which is expected to reduce the toxicity of the particles toward zebrafish.

In this study, zebrafish was selected as the model organism to study the 96-h acute toxicity of the CMC-SNs. The 96-h acute fish toxicity tests were based on the Organization for Economic Co-operation and Development (OECD) Test Guidelines. Seven concentrations (finally, five were selected) and one control group were used, with three replicates for each treatment. Zebrafish were considered dead if there was no visible movement when they were gently touched by the caudal peduncle. Silver nitrate and streptomycin were selected for comparison to CMC-SNs in the safety evaluation. In this experiment, CMC-SNs, silver nitrate and streptomycin were each added into 4 L separate transparent beakers. Then, 10 zebrafish of similar size and healthy state were put into the beaker. The beaker was changed with water and medicine every 24 h to keep the growth environment of zebrafish fresh. For the setting of drug concentration, a pre-experiment was performed to determine the appropriate concentration range.

In the individual toxicity tests of zebrafish, LC_50_, 95% confidence intervals (CI), R^2^ and chi-square were calculated by probit analysis using SPSS Statistics.

### 2.9. Cure of Rice Bacterial Diseases

Rice bacterial blight spreads through soil. In order to infect the rice plants, the bacterial suspension of 1 × 10^6^ CFU / mL was used to irrigate the potted rice soil. Each group was irrigated with 200 mL bacterial suspension twice during the growth period. Rice plants were cultured at around 28 °C and the occurrence of rice bacterial blight was observed every day. In the early stage of rice bacterial blight, CMC-SNs prepared in this paper were applied to the diseased leaves of rice at concentrations of 20, 10, 5 and 0 mg/L, respectively. The leaves were treated twice in total. The disease status of rice leaves was analyzed on the 14th day after inoculation.

Grading assessment method [22]:

Level 0: No leaf infection;

Level 1: The area of damaged leaf/the area of whole leaf < 0.2;

Level 3: The area of damaged leaf/the area of whole leaf = 0.25;

Level 5: The area of damaged leaf/the area of whole leaf = 0.5;

Level 7: The area of damaged leaf/the area of whole leaf = 0.75;

Level 9: The area of damaged leaf/the area of whole leaf = 1.

Disease index (DI) = $\frac{\Sigma (N_{n}\times G_{n})}{N\times G_{m}}\times 100N_{n}$ is the number of leaves with disease grade n, and $G_{n}$ is the number of disease grades; *N* is the total number of leaves investigated, and $G_{M}$ is the maximum number of disease grades.

Disease suppression efficiency =

$$\frac{{\mathrm{DI}}_{c}-{\mathrm{DI}}_{t}}{{\mathrm{DI}}_{c}}\times 100\%{\mathrm{DI}}_{c}$$

is the disease index of the control group, and $DI_{t}$ is the disease index of the treatment group.

## 3. Results and Discussion

### 3.1. Characterization of CMC-SNs

The CMC-SNs prepared in this paper are reduced in situ by CMC-Na under the induction of sunlight. The solution was changed from colorless to reddish brown. According to others’ work, the color change is due to the excitation of localized surface plasmon resonance (LSPR) [23]. This color depends on the size, shape, morphology, composition and dielectric environment of the prepared silver nanoparticles. The optical property was studied by UV-vis absorption spectrophotometry.

**Figure 1:**
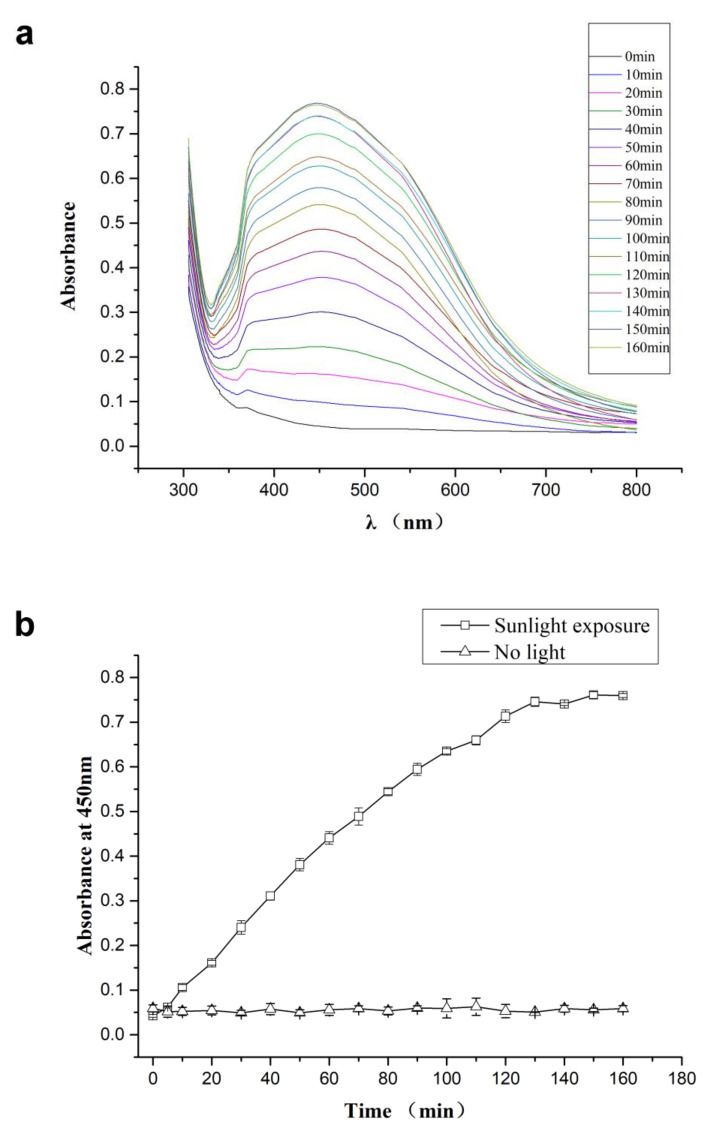
The UV-vis absorption of CMC-SNs (sodium-stabilized silver nanoparticles). (**a**) Absorption curves at different times. (**b**) The curve of time and absorption at 450 nm.

The timing starts from the dropping of silver nitrate into the system, and a small amount of solution is taken out every 10 min to test by UV-vis absorption spectrophotometry analysis. Different sizes of spherical metal nanostructures can absorb and reflect the light of different wavelengths. The LSPR absorption band of spherical AgNPs is usually around 450 nm. CMC-SNs contribute to the absorption band around 400–500 nm in the spectrum, and the process of reaction can be indicated by the intensity of the absorption peak (Figure 1). The 450 nm absorption peak of silver nanoparticles increased with reaction time (Figure 1a), and it was concluded from the data that the reaction was complete after around 150 min under these experimental conditions (Figure 1b).

**Figure 2:**
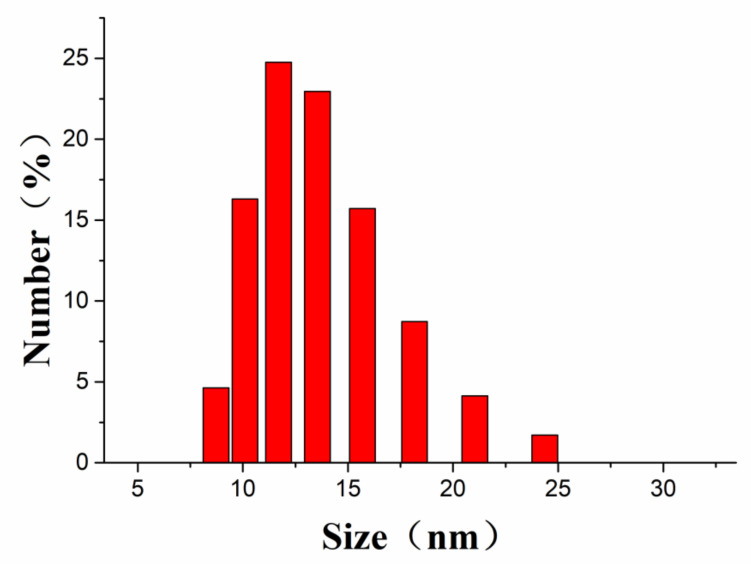
Particle size distribution of CMC-SNs by DLS (dynamic light scattering).

**Figure 3:**
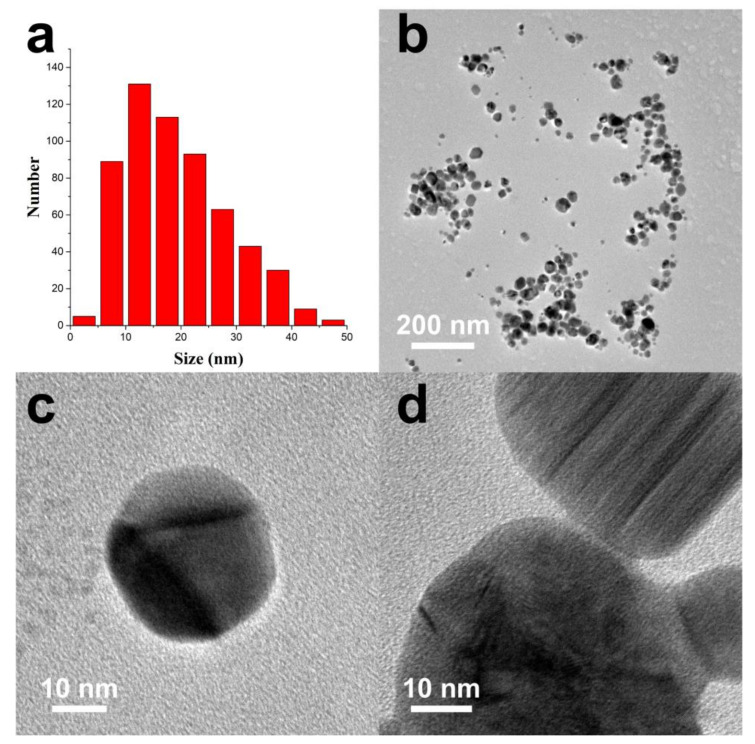
(**a**) particle size distribution of CMC-SNs; (**b**–**d**) TEM (transmission electron microscope) images of CMC-SNs.

**Figure 4:**
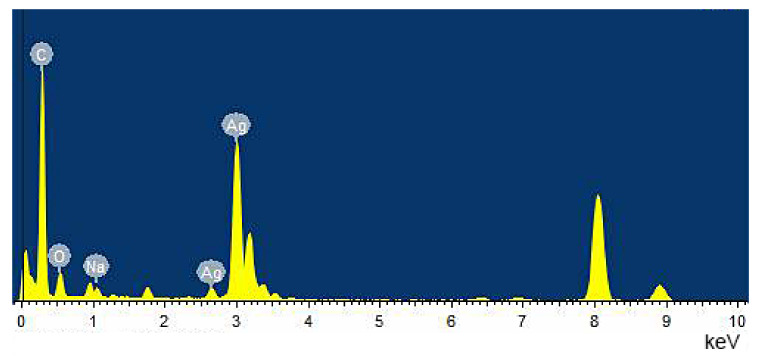
Energy-dispersive X-ray (EDS) spectrum of CMC-SNs. The peak at 8 keV is the copper element in the copper plate used in TEM sample preparation.

In this paper, silver nitrate was used as a starting material, and sodium carboxymethylcellulose was used as a stabilizer and template for sunlight-induced in situ reduction to prepare nano scale silver particles. The preparation process is a “green” or environmentally friendly, one-step synthesis with no toxic reagents. CMC-Na is a macromolecular polysaccharide derivative, with many negatively charged oxygens species in its structure, and it also has a spaced network structure. Therefore, CMC-Na is a good stabilizer and template for silver ions to build silver nanoparticles. Through the sunlight-induced reduction, silver nanoparticles with uniform particle size and similar shape are prepared. DLS was used to analyze the particle size distribution (Figure 2) and showed that the size of CMC-SNs was around 13.53 ± 4.72 nm. The morphology, size and dispersion of nano-silver particles were further studied by TEM (Figure 3b–d). Figure 3a shows the nanoparticle size (diameter) distributions, which were manually measured for 290 nanoparticles with ImageJ software [24]. The CMC-SNs prepared in this way were irregular spheres; in a dispersed state, 80% of the particles were distributed between 0 and 25 nm, which is consistent with the DLS data. In Figure 3c,d, an obvious lattice structure can be seen. Figure 4 shows the energy spectrum analysis (EDS), and the main elements besides Ag are C, O and Na, which are the components of CMC-Na.

**Figure 5:**
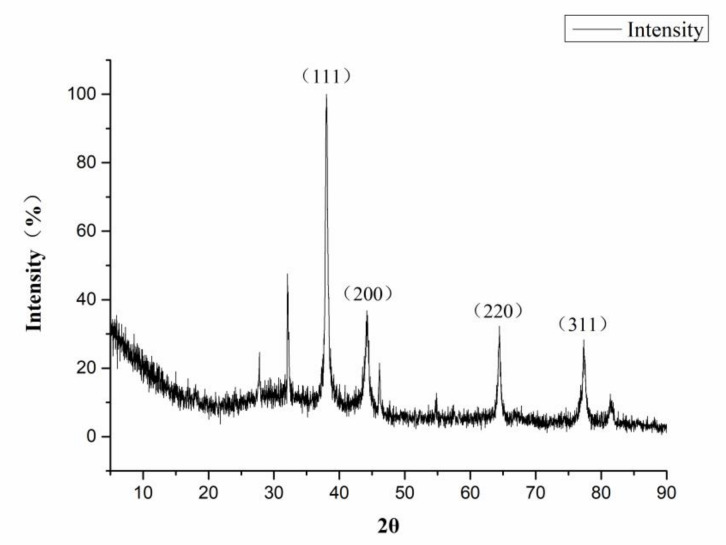
XRD pattern of CMC-SNs.

In Figure 5, XRD shows the lattice structure of the CMC-SNs. There are four obvious peaks at the 2θ angles of 38.1, 44.3, 64.4 and 77.4. Compared with the diffraction peaks of a standard silver element (PDF#04-0783), the lattice plane values are assigned to (111), (200), (220) and (311), which confirms that the crystal structure of silver in the particles is face-centered cubic. At the same time, the existence of a lattice structure can also be observed in the TEM image (Figure 3c,d).

### 3.2. Antibacterial Activity of CMC-SNs

The CMC-SNs prepared in this study demonstrate high antibacterial activity. Compared with antibiotics, CMC-SNs have high antibacterial activity toward rice bacterial blight. Further, the preparation of silver nanoparticles does not significantly reduce the bactericidal ability of the silver.

#### 3.2.1. Broth Dilution Method

Figure S1a shows the *Xoo* treated with CMC-SNs. The concentration gradient is 0, 0.03125, 0.0625, 0.125, 0.25, 0.5, 1, 2, 4 and 8 mg/L from left to right; the MIC is 1 mg/L. Figure S1b shows the *Xoo* treated with streptomycin. The concentration gradient from left to right is 0, 57.9, 69.4, 83.3, 100, 120, 144, 178.2, 207.4 mg/L, and the MIC is 178.2 mg/L. The CMC-SNs prepared in this study show much higher antimicrobial activity toward *Xoo* than streptomycin. It can be seen that streptomycin produces strong resistance in the control of rice bacterial blight at present because it has been used excessively. It is urgent to replace it with new drugs with have special mechanisms for strong antibacterial ability and low resistance.

Figure S1c shows the test results of treating *Xoo* with silver salts for 12 h. *Xoo* was treated with the same concentration of CMC-SNs, and the MIC was 0.125 mg/L. Silver nanoparticles may have reduced antibacterial ability in comparison to silver ions, but the MIC is still lower than the Streptomyces MIC (178.2 mg/L). This result is consistent with other researchers’ conclusions [5]. This work shows that the mechanism of silver ions is similar to that of AgNPs, and its antibacterial activity toward bacteria is slightly better than that of AgNPs.

**Figure 6:**
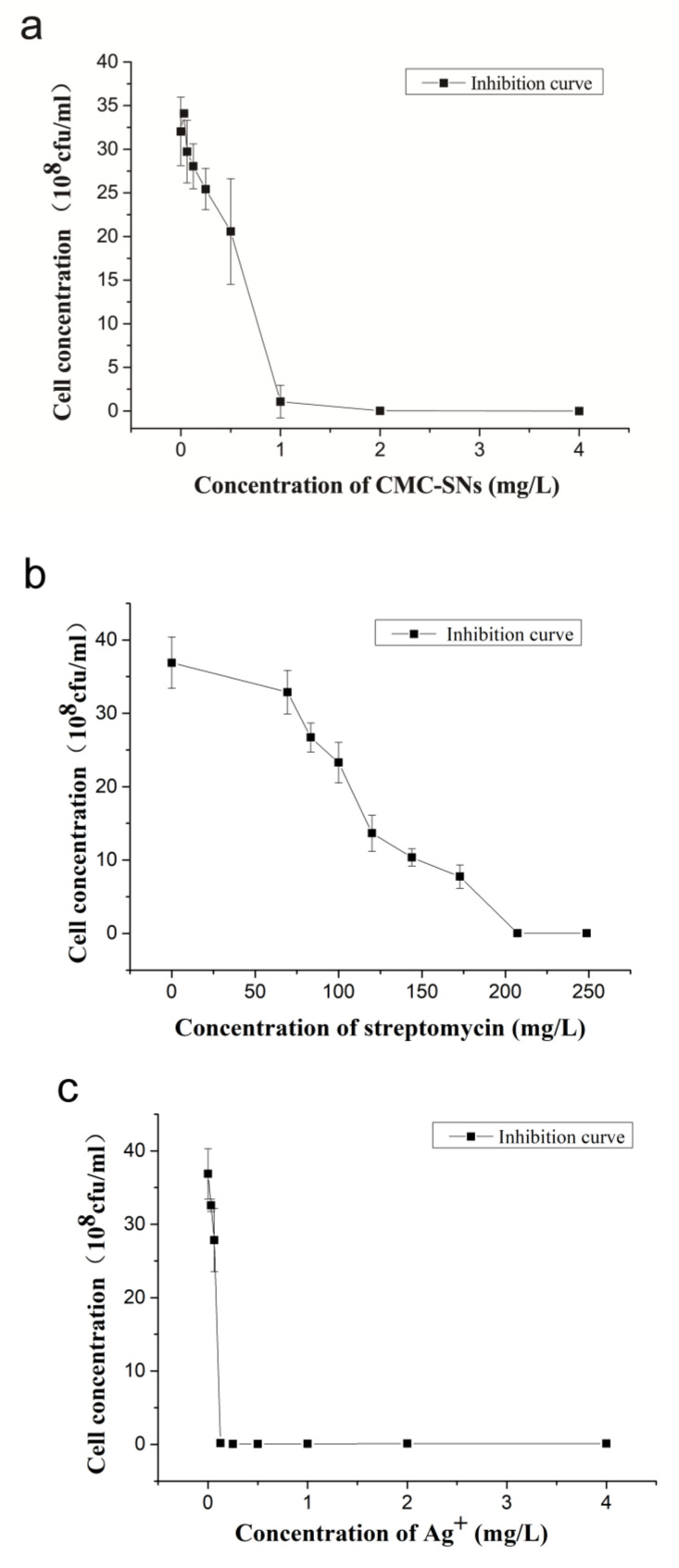
Bacterial cell concentration after 12 h of different treatments. (**a**) Treated with CMC-SNs; (**b**) treated with streptomycin; (**c**) treated with silver nitrate.

UV-vis spectroscopy was used to analyze the antibacterial activity of the above drugs, and the absorption value at 600 nm was taken as a reference [18]. Figure 6 shows the relationship between bacterial cell concentration and drug concentration.

#### 3.2.2. Broth Dilution Method

In order to show the antibacterial activity of CMC-SNs more intuitively, we used the plate colony count method to evaluate the bacterial survival (Figure S2), to obtain the MBC of the drug. The bactericidal ability of silver nitrate is slightly stronger than that of CMC-SNs, while that of streptomycin is much lower than that of CMC-SNs. Figure S2a shows the agar plate of the CMC-SNs. The treatment group of 1 mg/L bacteria was completely inhibited in broth culture medium, but there was still a colony found in the agar plate, so its MBC was 2 mg/L. Figure S2b shows the bacteriostatic streptomycin plate. In the broth medium, the 178.2 mg/L group showed that the growth of Xoo was completely inhibited, while a small number of bacteria survived in the agar plate, so the MBC of streptomycin to Xoo was 207 mg/L. Figure S2c shows the silver nitrate treatment group with MBC of 0.125 mg/L, which is consistent with the results in the broth medium.

Table 1 shows the MIC and MBC values of three agents to *Xoo*. The results show that the CMC-SNs prepared in this paper have high antibacterial activity against rice bacterial blight, while the bacteria have high resistance to streptomycin. Compared with silver ions, the antibacterial activity of CMC-SNs prepared by the method reported in this paper is slightly lower, which is consistent with similar results reported in the literature. However, it has been reported that the silver nanoparticles coated with polysaccharide have the characteristic of slow release [25], which may be more suitable for the control of rice bacterial diseases.

Table 2 shows the MIC values of CMC-SNs prepared in this paper for *P. syringae* pv. *Panici* (Elliott) Young et al. (ATCC19860) and *Pseudomonas syringae* pv. *syringae* Van Holl (ATCC19875). The MIC values for *P. syringae* pv. *Panici* (Elliott) Young et al. and *Pseudomonas syringae* pv. *syringae* Van Holl are 0.25 mg/L and 0.5 mg/L, respectively. The results of the broth medium are shown in Figures S4 and S5. These results suggest that CMC-SNs have a good antibacterial effect on other rice bacterial diseases.

### 3.3. Morphological Analysis

**Figure 7:**
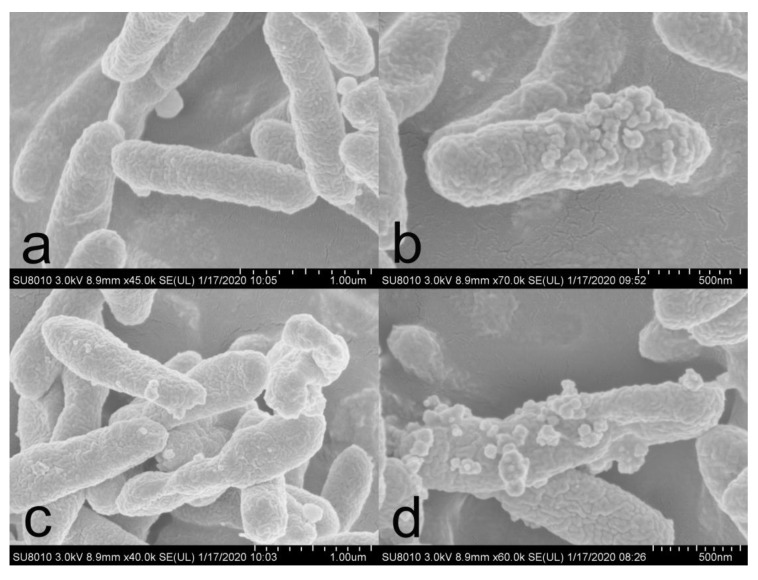
SEM of *Xoo.* (**a**) Control group; (**b**–**d**) treated by CMC-SNs.

SEM was used to observe the changes in the surface structure of the treated bacteria. As shown in Figure 7, the untreated bacterial cell wall is intact and smooth, while the treated bacterial surface clearly shows the attachment of nano-silver particles. The cell wall and cell membrane of bacteria in Figure 7c have been obviously damaged and are leaky, and there are clearly silver nanoparticles attached near the damaged area. Figure 7d shows that the bacterial cells have been destroyed and the intracellular substances have flowed out to the environment. These results strongly indicate that nano-silver can destroy the structure of the cell membrane and can lead to the leakage of substances in the cell.

### 3.4. Membrane Permeability Analysis

#### Fluorescent Dye Analysis

**Figure 8:**
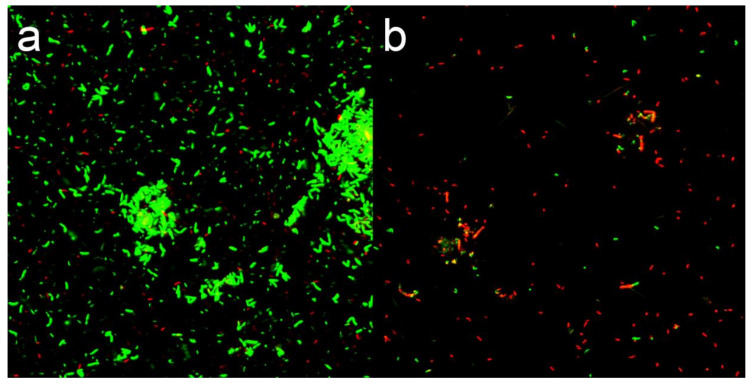
Bacterial fluorescence image treated by SYTO9/PI. (**a**) Control group; (**b**) treated by CMC-SNs for 12 h.

SYTO9/PI was used to stain the treated and untreated bacteria. The green fluorescence dye (SYTO9) can penetrate all bacterial cell membranes and stain the bacteria, while the red fluorescence dye (PI) can only enter into the damaged bacterial cell membrane and stain the nucleic acid material [26]. In Figure 8, the results showed that intense green fluorescence was produced by untreated bacteria after 12 h of culture, as well as a small amount of red fluorescence due to natural apoptosis. After 12 h of CMC-SN treatment, PI entered the bacterial cells and produced red fluorescence. The proportion of red fluorescence increased significantly over the signal observed for untreated bacteria, indicating that the bacterial cell membrane was indeed damaged by CMC-SNs. The results confirmed the conclusion of SEM that the bacterial cell membrane was damaged by CMC-SNs.

### 3.5. Analysis of Oxygen Free Radicals (ROS)

**Figure 9:**
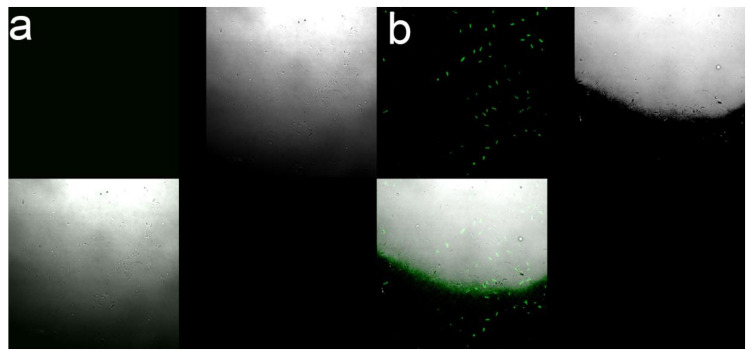
Bacterial fluorescence image treated by H2DCFDA. (**a**) Control group; (**b**) treated by CMC-SNs.

The production of ROS in bacteria was characterized by fluorescent dye H2DCFDA. ROS in bacteria is demonstrated by the presence of green fluorescence. If there is no ROS, there is no green fluorescent signal. In Figure 9, compared with brightfield, there was obvious green fluorescence in the bacterial cells of the treatment group, indicating that active oxygen radicals were produced in these bacteria, while there was no green fluorescence signal in the untreated group. These ROS are known to destroy the internal structure of bacteria, which may be the key to killing bacteria by silver nanoparticles.

### 3.6. Safety Evaluation

Nanomaterials have only recently begun to be used in agriculture, so it is necessary to know the interaction between nanomaterials and the environment clearly. In this paper, CMC-SNs were prepared for testing against rice bacterial diseases. Therefore, zebrafish was selected as a non-target organism to study the safety of CMC-SNs. We chose streptomycin and silver ion treatments as comparisons and the test results are shown in Figures S5–S7 and Table 3. Silver ions show high toxicity toward zebrafish; the LC_50_ of silver ions is 41.702 μg/L, and this result is similar to others’ work [27]. For CMC-SNs and streptomycin, when the concentration reached 100 mg/L, there was no sign of zebrafish death; it can be concluded that CMC-SNs have no acute toxicity toward zebrafish.

### 3.7. Cure of Rice Bacterial Diseases

**Figure 10:**
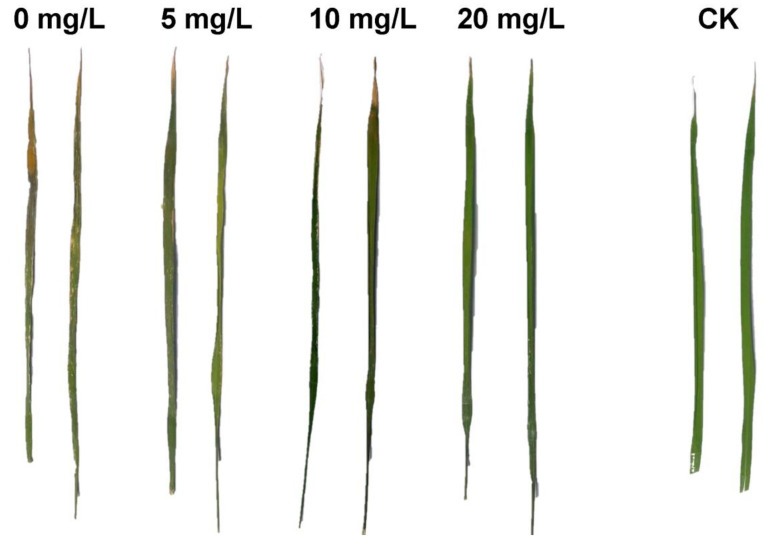
Effect of CMC-SNs on cure of rice bacterial blight.

Figure 10 and Table 4 show the results of the cure of rice bacterial blight by CMC-SNs. The results show that rice bacterial blight could be well inhibited in the early stage of the disease, thus preventing the rice leaves from withering and dying. When the CMC-SN concentration was 5 and 10 mg/L, rice bacterial wilt was inhibited by 54.7% and 60.8%, respectively, and the disease index was obviously lower than that of the 0 mg/L group. The occurrence of rice bacterial blight was clearly inhibited at a lower concentration. When the concentration reached 20 mg/L, rice bacterial blight was completely inhibited, and the disease inhibition rate reached 100%.

## 4. Conclusions

In this paper, a simple and green chemical method was used to prepare CMC-SNs. The size distribution of CMC-SNs prepared by this method is around 15 nm, which has good dispersion and a uniform shape. Compared with other studies [15,16,28,29], the synthesis method of CMC-SNs prepared in this paper is green and entails low cost and safe operation, providing a new method for the application of silver nanoparticles in agricultural production. The results of bioassays show that the CMC-SNs have high antibacterial activity against the pathogenic bacteria of rice bacterial blight, *Pseudomonas avenae* Manns and rice bacterial sheath rot. This can also be demonstrated by comparing others’ work [15,30]. The bacteriostatic mechanism of *Xoo* was explored. Firstly, silver particles acted on the bacterial cell wall to damage the cell wall and change the permeability of the cell membrane and finally cause the cell to leak. However, silver particles entering the bacteria can produce ROS to destroy the internal structure of cells, which kills the bacteria. In view of the impact of nanoparticles on the environment, the acute toxicity of zebrafish was studied. The results show that CMC-SNs have no acute toxicity toward zebrafish, while silver ions show acute toxicity toward zebrafish. According to the research, the biosafety of silver nanoparticles depends on its coat, impurities and particle size [21]. The CMC-SNs prepared in this paper have better biological safety than others [27]. Therefore, CMC-SNs may be safer for aquatic organisms in a rice field environment. In the cure experiment of rice bacterial blight, the CMC-SNs prepared in this paper could obviously inhibit the occurrence of the disease and could be used in the control of rice bacterial blight under the experimental conditions. The bactericidal activity and biological toxicity of silver nanoparticles often increase or decrease at the same time, depending on the size of the particles [9]. The CMC-SNs prepared in this paper not only have high bactericidal activity but also have certain biological safety. The CMC-SNs prepared by this method are environmentally friendly, safe to non-target organisms and have high antibacterial activity and the ability to control rice bacterial diseases. CMC-SNs can be used to replace streptomycin and other highly microbial-resistant bactericides to become a new and efficient pesticide for the control of rice bacterial diseases.

The future directions of silver nanoparticles used in pesticides will be greater biosafety, higher bioactivity and controlled release. Based on this work, we will focus on more biosafety studies in the future and attempt to change the particle coatings in order to ensure slower and more accurate release.

## Figures and Tables

**Table 1:** MIC (minimum inhibitory concentration) and MBC (minimum bactericidal concentration) of silver nitrate, CMC-SNs and streptomycin against *Xoo.*

*Xoo*	MIC (mg/L)	MBC (mg/L)
Silver nitrate	0.125	0.125
CMC-SNs	1	2
Streptomycin	178.2	207

**Table 2:** MIC of CMC-SNs against *P. syringae* pv. panici (Elliot) Young et al. and *Pseudomonas syringae* pv. *syringae* Van Holl.

Bacterial Diseases	Pathogenic Bacteria	MIC (mg/L)
*Pseudomonas avenae* Manns	*P. yringae* pv. *panici* (Elliott) Young et al.	0.25
Rice bacterial sheath rot	*Pseudomonas syringae* pv. *syringae* Van Holl	0.5

**Table 3:** LC_50_ values of silver nitrate, CMC-SNs and streptomycin to zebrafish.

Test Drugs	Silver Nitrate	CMC-SNs	Streptomycin
Exposure (h)	96	96	96
LC_50_	41.702 μg/L	>100 mg/L	>100 mg/L
R^2^	0.959	-	-
Chi-square	7.388	-	-
95% confidence interval	(39.601–43.694)	-	-

**Table 4:** Disease index and disease suppression efficiency of rice bacterial blight.

Treatment	Disease Index (DI)	Disease Suppression Efficiency (%)
CK (control check)	0	-
0 mg/L	61.1	0
5 mg/L	27.7	54.7
10 mg/L	20	60.8
20 mg/L	0	100

## Supplementary Materials

The following are available online at https://www.mdpi.com/2079-4991/10/10/2007/s1, Figure S1: Broth medium after 12 h of different treatments. Figure S2: Agar plate of different treatments. Figure S3: Broth medium of *Pseudomonas syringae* pv. *syringae* Van Holl after 12 h of CMC-SNs treatments. Figure S4: Broth medium of *P. syringae* pv. *panici* (Elliott) Young et al. after 12 h of CMC-SNs treatments. Figure S5: 96 h acute toxicity test of zebrafish treated with silver nitrate. Figure S6: 96 h acute toxicity test of zebrafish treated with CMC-SNs. Figure S7: 96 h acute toxicity test of zebrafish treated with streptomycin.
