# Supplementary material for: Sunlight-Induced Synthesis of Non-Target Biosafety Silver Nanoparticles for the Control of Rice Bacterial Diseases
Source: Nanomaterials (Basel). 2020 Oct 12;10(10):2007. doi: 10.3390/nano10102007 (PMC7600880; doi:10.3390/nano10102007)
Supplement: Supplementary file 1 [file nanomaterials-10-02007-s001.pdf]

nanomaterials

Supplementary Materials

# Sunlight-Induced Synthesis of Non-Target Biosafety Silver Nanoparticles for the Control of Rice Bacterial Diseases

**Hongyi Shang, Zehao Zhou, Xuemin Wu * , Xuefeng Li * and Yong Xu ***

Innovation Center of Pesticide Research, Department of Applied Chemistry, College of Science, China Agricultural University; hongyishang@cau.edu.cn (H.S.); zhouzh@nanoctr.cn (Z.Z.)

* Correspondence: wuxuemin@cau.edu.cn (X.W.); 91030@cau.edu.cn (X.L.); cauxy@cau.edu.cn (Y.X.); Tel.: +86-010-6273-2961 (X.W.); +86-010-6273-3924 (X.L.); +86-010-6273-4645 (Y.X.)

Antibacterial activity against *Xanthomonas oryzae pv.oryzae, P.yringae* pv.*panici* （*Elliott*） *Young et a1* and *Pseudomonas syringae pv.syringae Van Holl*

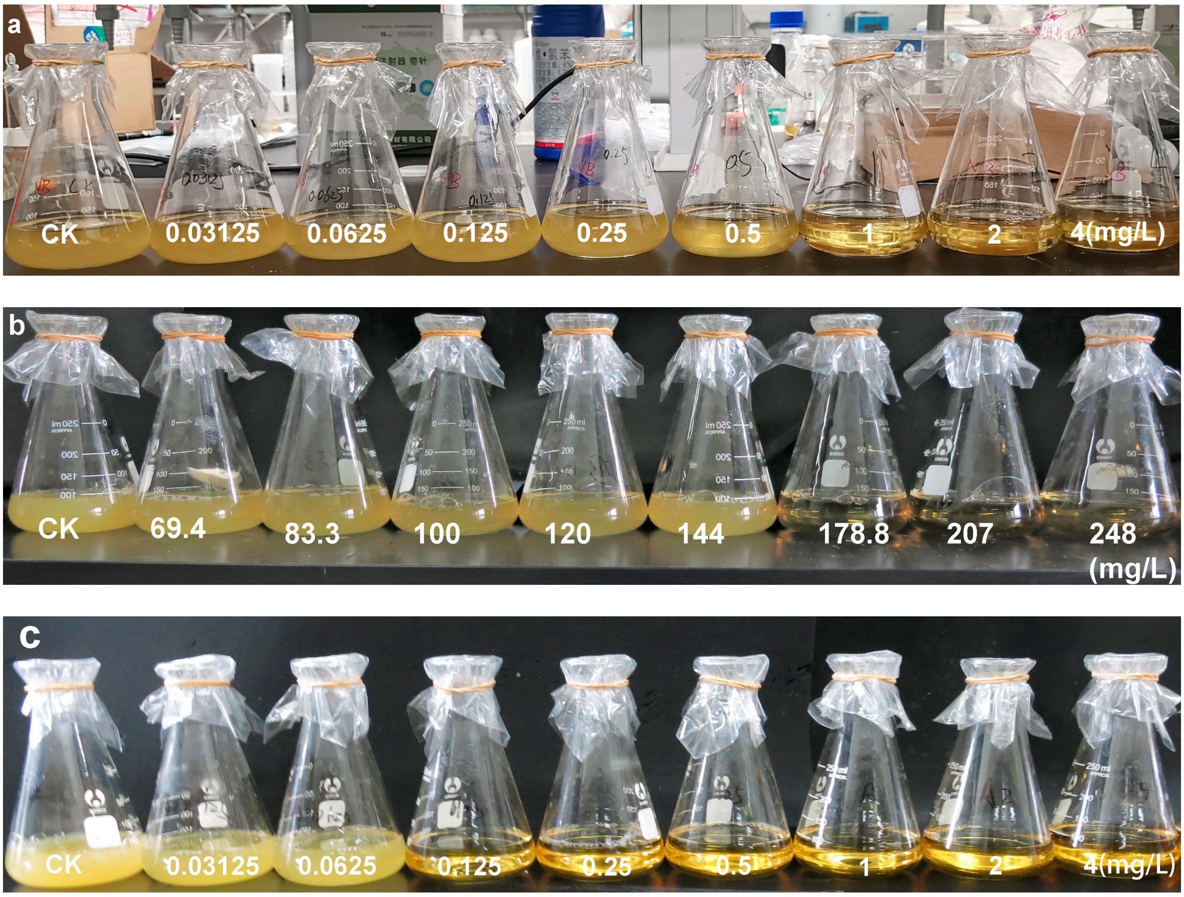


**Figure S1.** Broth medium after 12 hours of different treatments. (**a**) Treated with CMC-SNs; (**b**) Treated with streptomycin; (**c**) Treated with silver nitrate.

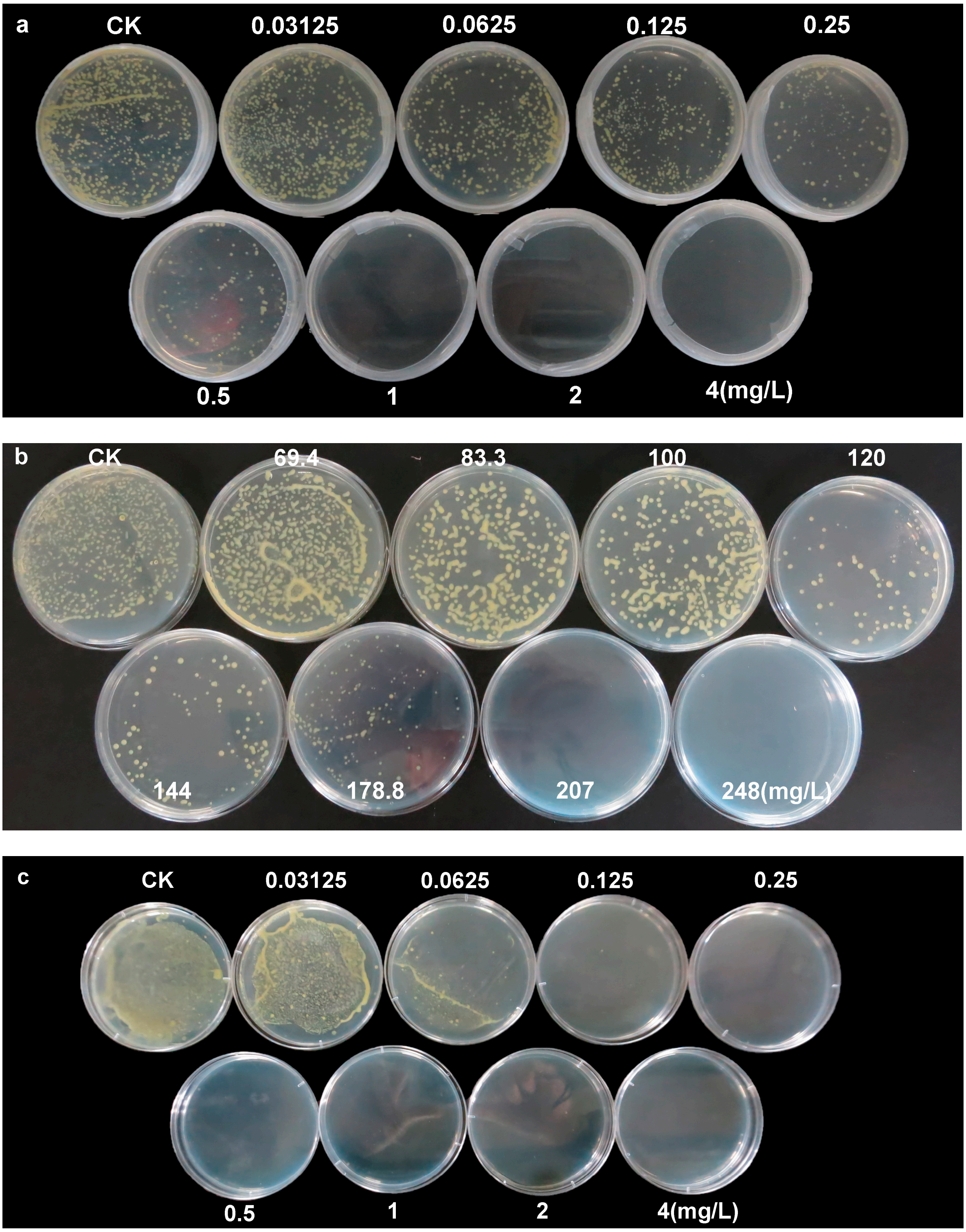


**Figure S2.** Agar plate of different treatments. (**a**) Treated with CMC-SNs;(**b**) Treated with streptomycin;(**c**) Treated with silver nitrate.

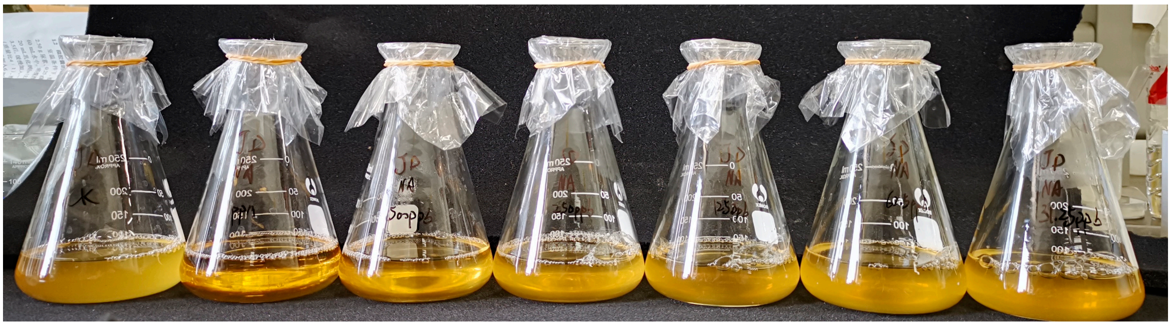

**Figure S3.** Broth medium of *Pseudomonas syringae* pv.*syringae Van Holl* after 12 hours of CMC-SNs treatments. The concentration is 0 mg/L,1 mg/L,0.5 mg/L,0.25 mg/L,0.125 mg/L,0.0625 mg/L,0.03125 mg/L from left to right.

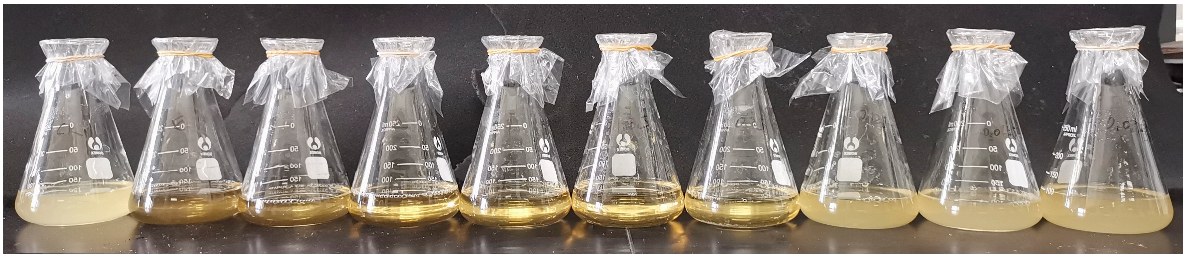

**Figure S4.** Broth medium of *P.yringae pv.panici*（*Elliott*）*Young et a1* after 12 hours of CMC-SNs treatments. The concentration is 0 mg/L,8 mg/L,4 mg/L,2 mg/L,1 mg/L,0.5 mg/L,0.25 mg/L,0.125 mg/L,0.0625 mg/L,0.03125 mg/L from left to right.

Acute toxicity test of zebrafish

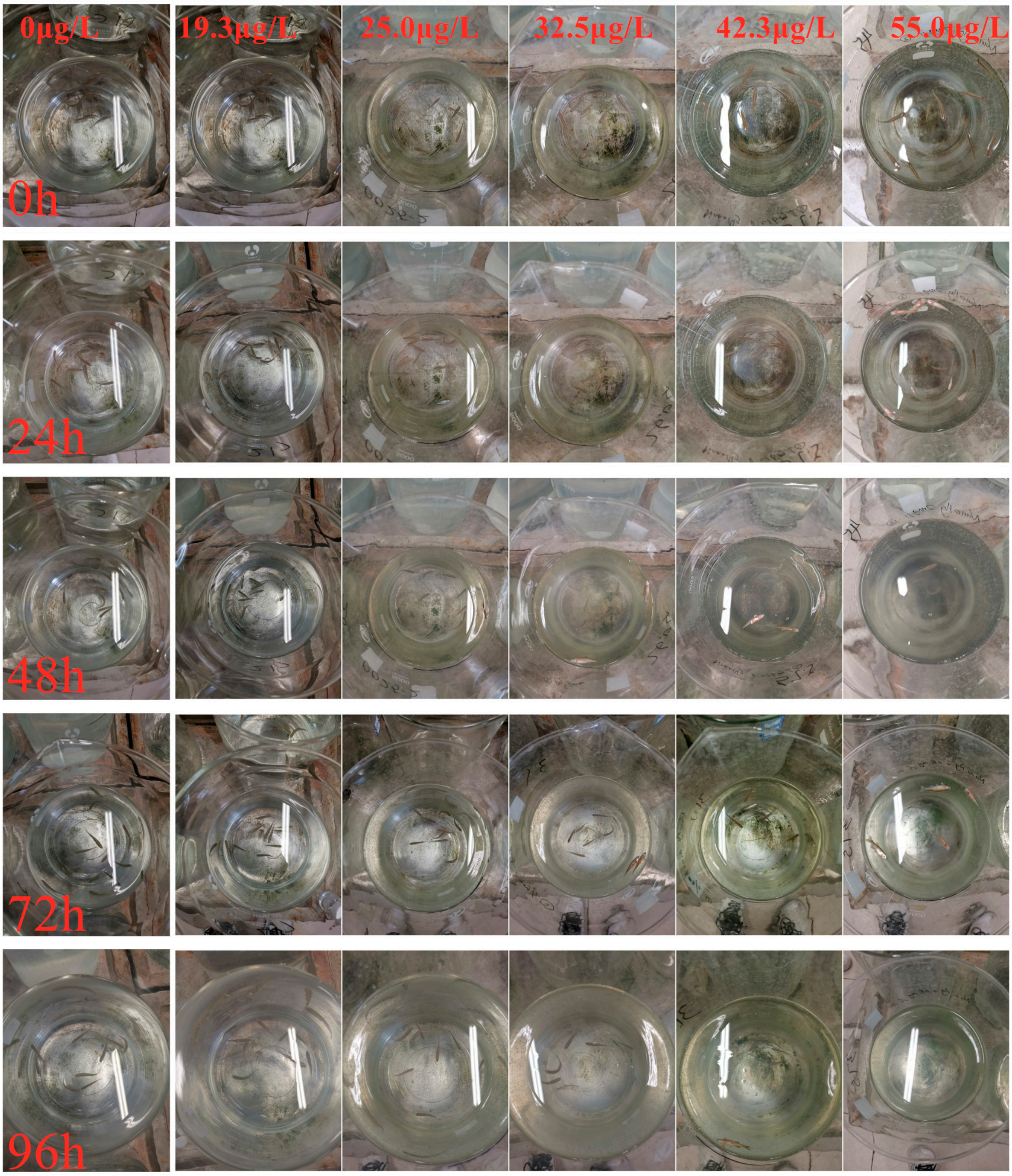


**Figure S5.** 96 h acute toxicity test of zebrafish treated with silver nitrate.

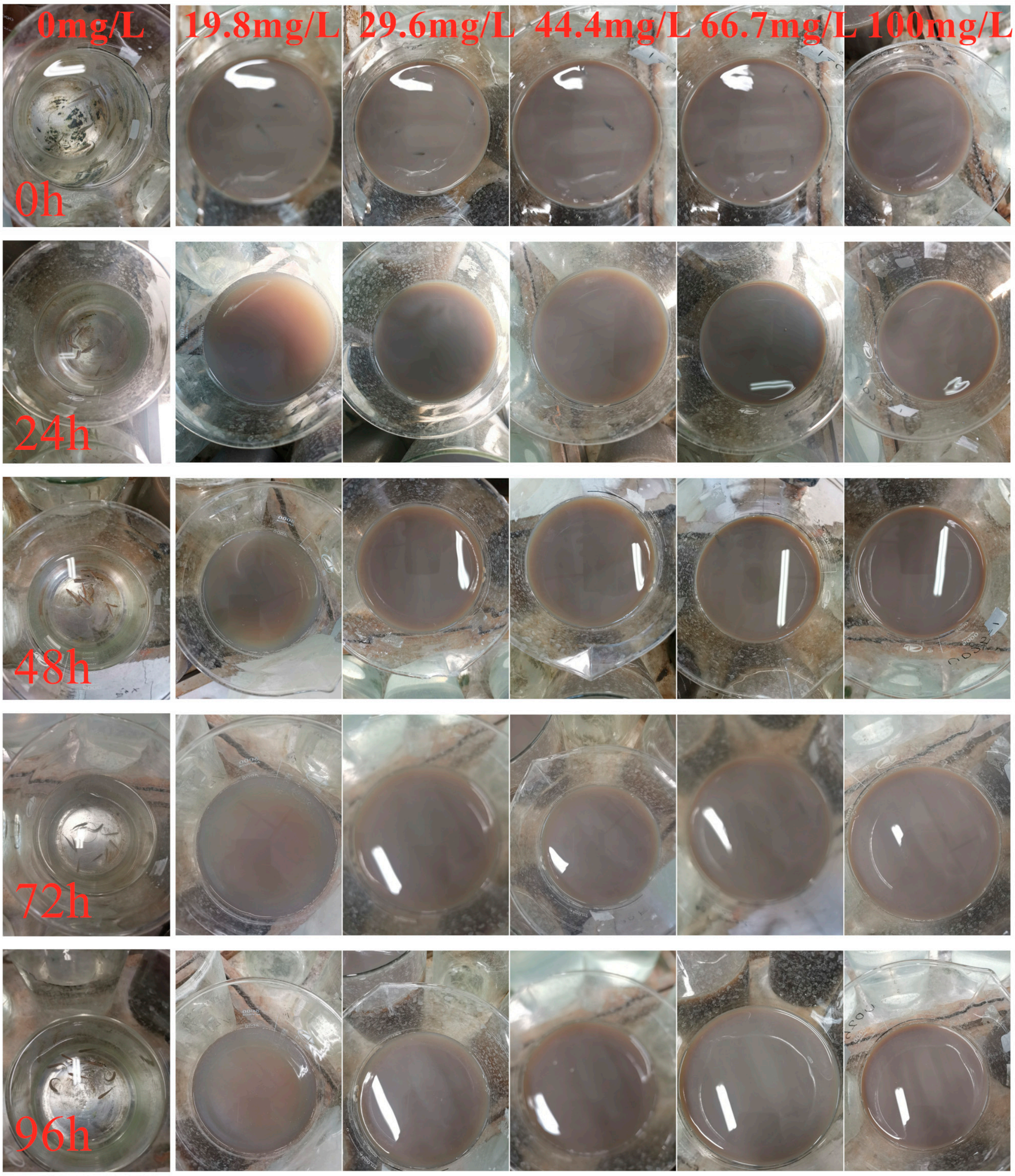


**Figure S6.** 96 h acute toxicity test of zebrafish treated with CMC-SNs.

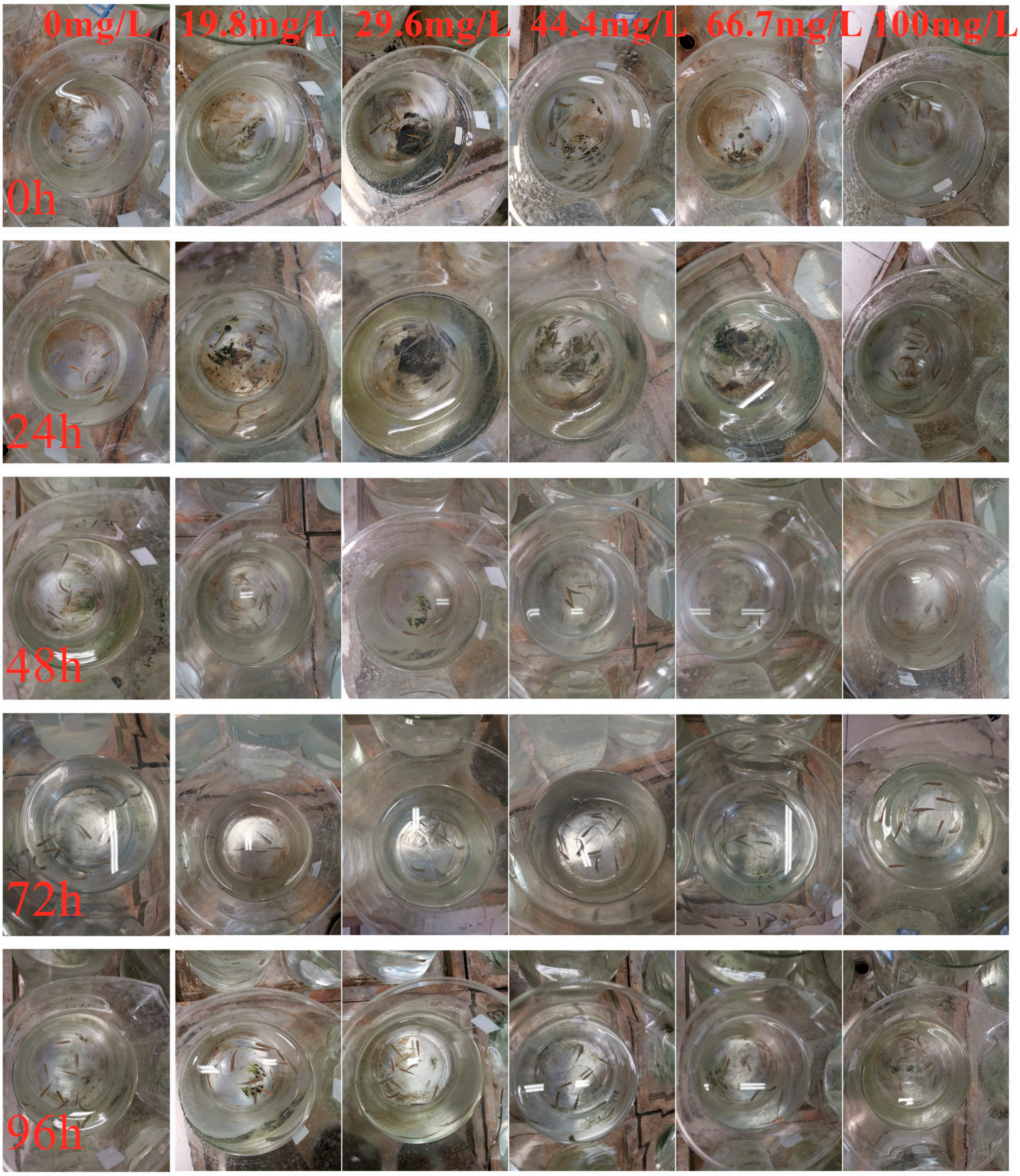


**Figure S7.** 96 h acute toxicity test of zebrafish treated with streptomycin.

© 2020 by the authors. Submitted for possible open access publication under the terms and conditions of the Creative Commons Attribution (CC BY) license (http://creativecommons.org/licenses/by/4.0/).
